# Changes in renal papillary density after hydration therapy in calcium stone formers

**DOI:** 10.1186/s12894-018-0415-7

**Published:** 2018-11-12

**Authors:** Pietro Manuel Ferraro, Matteo Vittori, Giuseppe Macis, Alessandro D’Addessi, Gianmarco Lombardi, Claudia Palmisano, Jacopo Gervasoni, Aniello Primiano, Pier Francesco Bassi, Giovanni Gambaro

**Affiliations:** 1 U.O.C. Nefrologia, Fondazione Policlinico Universitario A. Gemelli IRCCS, Roma, Italia; 2 U.O.C. Clinica Urologica, Fondazione Policlinico Universitario A. Gemelli IRCCS, Roma, Italia; 3 U.O.C. Radiologia, Fondazione Policlinico Universitario A. Gemelli IRCCS, Roma, Italia; 4 U.O.C. Biochimica Clinica, Fondazione Policlinico Universitario A. Gemelli IRCCS, Roma, Italia; 5 Università Cattolica del Sacro Cuore, Roma, Italia

**Keywords:** Kidney stones, CT scan, Randall’s plaque, Hydration therapy

## Abstract

**Background:**

Previous studies have shown that, compared with non-stone formers, stone formers have a higher papillary density measured with computer tomography (CT) scan. The effect of increased hydration on such papillary density in idiopathic calcium stone formers is not known.

**Methods:**

Patients with recurrent calcium oxalate stones undergoing endourological procedures for renal stones at our Institution from June 2013 to June 2014 were considered eligible for enrolment. Enrolled patients underwent a baseline unenhanced CT scan before the urological procedure; after endoscopic removal of their stones, the patients were instructed to drink at least 2 L/day of a hypotonic, oligomineral water low in sodium and minerals (fixed residue at 180 °C < 200 mg/L) for at least 12 months. Finally, the patients underwent a follow-up unenhanced CT scan during hydration regimen.

**Results:**

Twenty-five patients were prospectively enrolled and underwent baseline and follow-up CT scans. At baseline, mean papillary density was 43.2 ± 6.6 Hounsfield Units (HU) (43.2 ± 6.7 for the left kidney and 42.8 ± 7.1 HU for the right kidney). At follow-up and after at least 12 months of hydration regimen, mean papillary density was significantly reduced at 35.4 ± 4.2 HU (35.8 ± 5.0 for the left kidney and 35.1 ± 4.2 HU for the right kidney); the mean difference between baseline and follow-up was − 7.8 HU (95% confidence interval − 10.6 to − 5.1 HU, *p* < 0.001).

**Conclusions:**

Increased fluid intake in patients with recurrent calcium oxalate stones was associated with a significant reduction in renal papillary density.

**Trial registration:**

NCT03343743, 15/11/2017 (Retrospectively registered).

## Background

Kidney stones are an increasingly common condition, with an estimated prevalence in the United States of about 10%; [1] similar figures have been reported in European populations. [2] This condition is characterized by a relatively high frequency of recurrence [3] and elevated direct and indirect costs. [4] Kidney stones are most commonly composed of calcium; [5] a frequently seen lesion in calcium stone formers is the presence of suburothelial deposits of calcium phosphate known as Randall’s plaques, which are thought to be the precursor lesion of idiopathic calcium oxalate stones. [6] Previous studies have shown that, compared with non-stone formers, stone formers have a higher papillary density measured with computer tomography (CT) scan, [7–11] likely reflecting the amount of calcium deposition within the renal parenchyma; it has also been suggested that papillary density values might correlate with first incident stones [12] and recurrent stones [13]. To date however, the effect of increased hydration on such papillary density in idiopathic calcium stone formers is not known. In our study, we analyzed changes in papillary density in recurrent idiopathic calcium stone formers before and after recommendations to increase fluid intake following urological stone removal.

## Methods

Patients undergoing endourological procedures for renal stones at our institution from June 2013 to June 2014 were considered eligible for enrolment. Inclusion criteria were recurrent stone disease and calcium oxalate stones (> 50% of the stone made of calcium oxalate). Stone composition was determined by Fourier transform infrared spectroscopy. Exclusion criteria were systemic conditions causing stones (hyperparathyroidism, intestinal malabsorption, renal tubular acidosis) and non-calcium oxalate stones. After signing written informed consent, patients underwent a baseline unenhanced CT scan before the urological procedure; the CT scans were performed using General Electric, Siemens, Philips and Toshiba equipment with a tube voltage variable from 120 to 140 kV, 80–643 mA and slices collimation from 1 to 5 mm. After endoscopic removal of their stones, the patients were instructed to drink at least 2 L/day of a hypotonic, oligomineral water low in sodium and minerals and rich in bicarbonate (fixed residue at 180 °C < 200 mg/L) for at least 12 months (Table 1). During follow-up, patients were contacted by phone every 3–4 months in order to maintain their adherence to increased hydration; at the first follow-up phone contact, patients were also asked to quantify the amount of fluid increase in terms of glasses per day (a glass would correspond to about 200 mL of water). Finally, the patients underwent a follow-up unenhanced CT scan during hydration regimen; this second CT was performed using similar technical specifications to the baseline CT scan. At that time point, blood and 24-h urine samples were also collected. Renal papillary density values were evaluated on baseline and follow-up CT scans and recorded as Hounsfield Unit (HU) values.

**Table 1:** Composition of the study water

Sodium	4.7
Calcium	57
Magnesium	3.5
Potassium	0.5
Bicarbonate	178
Fixed residue at 180 °C	178
pH	7.47

All values expressed as mg/L except for pH

Six papillae were measured in each renal unit by randomly choosing two papillae from each of the major renal calyces (upper, middle and lower), as such that the densities of 12 renal papillae per patient were measured, and their means calculated. The images were magnified to 5× to prevent contamination of the region of interest with the fat in the renal sinus. The measurements of the renal papillae were made taking an area of 10 mm^2^.

All urine and serum analytes were measured in the central laboratory of Fondazione Policlinico Universitario Gemelli IRCCS on a Siemens ADVIA chemistry XPT with the following exceptions: urine citrate and oxalate were measured with LTA kit for enzymatic colorimetric determination.

### Statistical analysis

Continuous variables were reported as means and standard deviations; categorical variables as frequencies and percentages. Arithmetic means of right and left renal papillary density measurements for each patient were calculated and used for statistical analysis. Differences between baseline and follow-up papillary density values were analyzed with paired t-tests; correlations between continuous variables were analyzed with Spearman tests. A *p*-value < 0.05 was considered statistically significant. Statistical analyses were performed with Stata 13.1 (StataCorp, TX, USA).

## Results

**Fig. 1:**
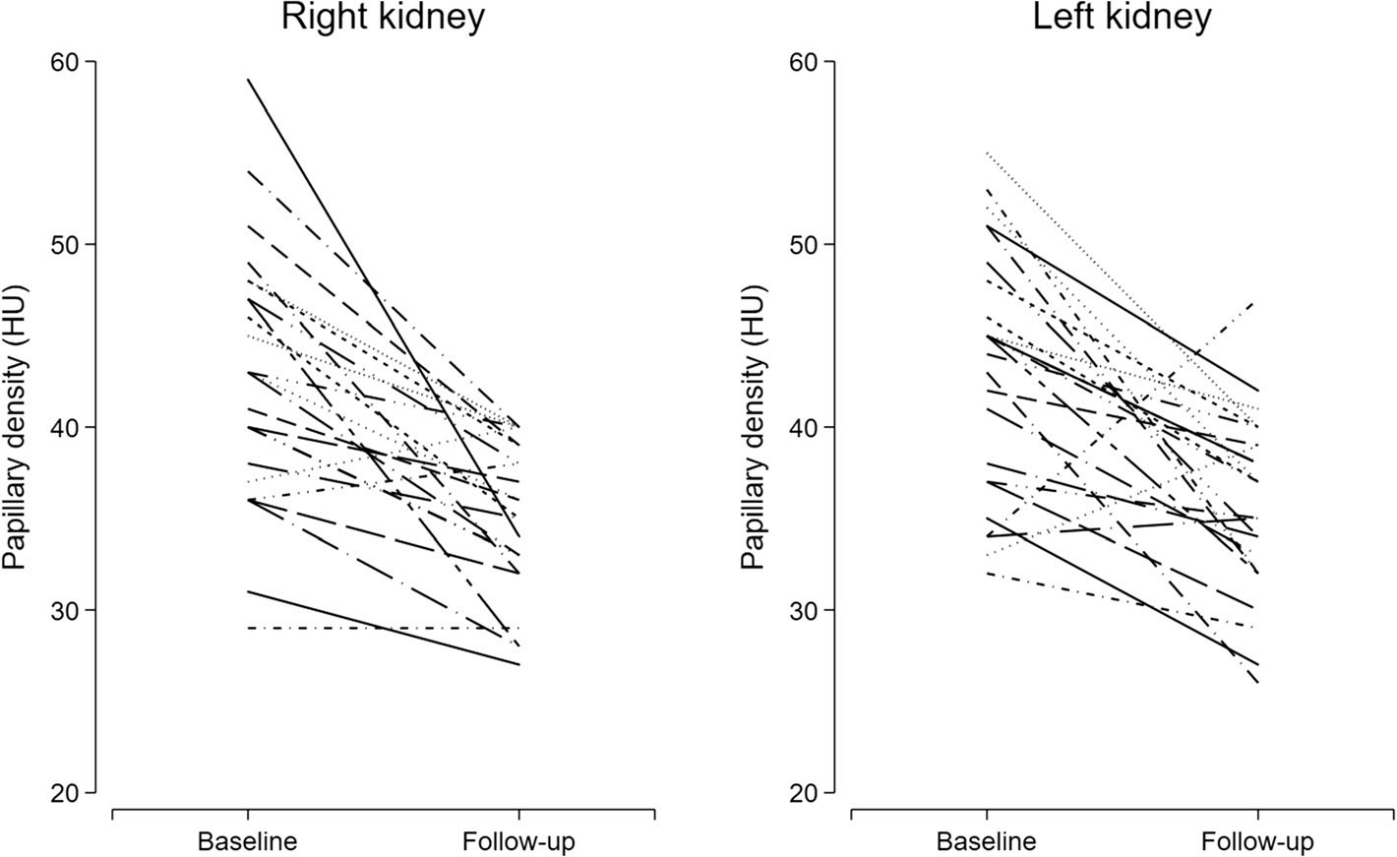
Individual changes in renal papillary density before and after hydration

Twenty-five patients were prospectively enrolled and underwent baseline and follow-up CT scans. Mean age at enrollment was 57 ± 17 years (range 18 to 78 years), 20 (80%) were males; average follow-up time was 22 ± 5 months (range 13 to 32 months); 22 patients received ureteroscopy and 3 percutaneous nephrolithotomy. All patients underwent measurements of papillary density on both kidneys except for one patient who had measurements taken only on the right kidney. At baseline, mean papillary density was 43.2 ± 6.6 HU (43.2 ± 6.7 for the left kidney and 42.8 ± 7.1 HU for the right kidney). At follow-up and after at least 12 months of hydration regimen, mean papillary density was significantly reduced at 35.4 ± 4.2 HU (35.8 ± 5.0 for the left kidney and 35.1 ± 4.2 HU for the right kidney); the mean difference between baseline and follow-up was − 7.8 HU (95% confidence interval − 10.6 to − 5.1 HU, *p* < 0.001). Individual changes in papillary density are represented in Fig. 1. The median self-reported increase in fluid intake during follow-up was 3 glasses (range 0 to 5 glasses). Blood and 24-h urine measurements performed at the time of follow-up CT scan are reported in Table 2. There was no correlation between the absolute change in papillary density and any of the laboratory measurements.

**Table 2:** Blood and 24 h urine chemistries

Blood	
Creatinine (mg/dL)	0.98 (0.23)
Calcium (mg/dL)	9.9 (0.3)
Phosphate (mg/dL)	3.4 (0.8)
Magnesium (mg/dL)	2.1 (0.1)
Sodium (mEq/L)	141 (2)
Potassium (mEq/L)	4.6 (0.7)
24 h urine	
Volume (mL/24 h)	2005 (765)
Creatinine (mg/24 h)	786 (340)
Calcium (mg/24 h)	98 (58)
Citrate (mg/24 h)	459 (208)
Oxalate (mg/24 h)	19 (13)
Uric acid (mg/24 h)	425 (192)
Phosphate (mg/24 h)	439 (164)
Magnesium (mg/24 h)	56 (27)
Sodium (mEq/24 h)	83 (30)
Potassium (mEq/24 h)	31 (12)

Data are expressed as means (standard deviation)

## Discussion

In our study, we found that a course of hydration with oligomineral water after urological procedures of stone removal was associated with a significant reduction in renal papillary density as assessed with CT scan in a group of patients with recurrent calcium oxalate stones.

Several studies have investigated the association between kidney stones and increased renal papillary density. [7–11] Most studies found that the higher papillary density in stone formers is similar in affected and unaffected kidneys; it also seems to be independent of stone composition. [14] A few, retrospective, studies have also linked higher papillary density to higher likelihood of developing a first stone [12], as well as recurrent stones [13]. Only one study looked at the relationship between papillary density and the urinary metabolic profile, particularly urinary calcium excretion, and found no association, [11] which is consistent with our findings of a lack of association between changes in papillary density and blood and urine chemistries. Average HU values in previous studies ranged from 37 to 54 for stone formers and from 21 to 37 for controls, with some degree of heterogeneity likely explained by different study populations. Baseline HU values in our study (43.2 HU) were similar to those reported for stone formers in previous studies (average 42.1 HU, range 35.6 to 54.4 HU); [7–11] interestingly, follow-up HU values in our study (35.4 HU) were closer to the range of HU values previously reported for non-stone formers in those studies (average 29.5 HU, range 21.0 to 36.6 HU).

An adequate intake of fluids is regarded as one of the mainstays of treatment for stone disease; [15, 16] a randomized controlled trial on patients at their first episode of idiopathic calcium stone showed that increasing water intake to obtain 2 L of urine per day or more reduced the risk of recurrence at 5 years by about 55%. [17] Despite the proven efficacy of hydration in stone disease, changes in renal papillary density associated with increased water intake have not been investigated before. The results of our study suggest that increased fluid intake is likely to modify the concentration of lithogenic salts in the renal tissue; whether these changes would reflect in an actual reduction in risk of recurrence remains to be elucidated. Our study also highlights the need to include the HU of the renal papillae in future studies investigating interventions for stone prevention.

Our study has several strengths: a cohort composed of phenotypically homogeneous patients (recurrent calcium oxalate stone formers), whose stone composition was assessed by means of the gold-standard infrared spectroscopy; [18] all patients underwent the same urological procedure and the radiological images were obtained and analyzed prospectively; they all received the same indication in terms of hydration (e.g., at least 2 L per day of hypotonic oligomineral water) and the follow-up was relatively long (at least 12 months); furthermore, patients were asked and reminded about their increased fluid intake through phone contacts. We also had available data on blood and 24 h urine chemistries.

Our study also has limitations. First, we did not have a control group; however, it would not have been ethical nor feasible to not recommend increased hydration after urological removal of stones. Second, follow-up time was not identical for all participants, as it was performed on routine clinical follow-up visits based on the clinicians’ judgement; however, all participants had at least 12 months of follow-up before the follow-up CT scan was performed. Third, adherence to the recommended fluid regimen was not assessed through changes in urine volume; however, the urine volume at follow-up was on average greater than 2 L, a figure higher than expected without intervention in a group of recurrent stone formers and thus likely increased compared with baseline. Fourth, we did not have a mean to assess long-term hydration habits of the patients. Fifth, the radiologist who assessed the HU value was not blinded to the patient status; this was done in order to allow the radiologist to sample the same ROI in baseline and follow-up CT scans. Finally, the documented changes in renal papillary density could not necessarily translate into reduced risk of recurrence, despite initial evidence for a predictive role of renal papillary density in stone development; future studies are warranted to investigate whether renal papillary density and changes thereof are independent predictors of incident stone formation.

## Conclusions

Increased fluid intake in patients with recurrent calcium oxalate stones is associated with a significant reduction in renal papillary density.

## Abbreviations

CT: Computer tomography
HU: Hounsfield Units
